# Supplementary material for: End Sequence Analysis Toolkit (ESAT) expands the extractable information from single-cell RNA-seq data
Source: Genome Res. 2016 Oct;26(10):1397–410. doi: 10.1101/gr.207902.116 (PMC5052061; doi:10.1101/gr.207902.116)
Supplement: Supplemental Material [file supp_gr.207902.116_Supp_Materials.tar.gz › ESAT-esat_v0.1/doc/html/classumms_1_1core_1_1utils_1_1_annotation_utils-members.html]

ESAT: Member List


ESAT


- Main Page
- Related Pages
- Packages
- Classes
- Files

- Class List
- Class Index
- Class Hierarchy
- Class Members

All Classes Namespaces Files Functions Variables Enumerator Pages

- umms
- core
- utils
- AnnotationUtils

umms.core.utils.AnnotationUtils Member List

This is the complete list of members for umms.core.utils.AnnotationUtils, including all inherited members.

|  |  |  |
| --- | --- | --- |
| collapseOverlappers(Map< String, Collection< Gene >> genes, boolean ignoreStrand) | umms.core.utils.AnnotationUtils | static |
| getChildren(T parent, Map< String, Collection< T >> children) | umms.core.utils.AnnotationUtils | static |
| getLargestParent(T child, Map< String, Collection< T >> parents) | umms.core.utils.AnnotationUtils | static |
| getMidpoint(Annotation annot) | umms.core.utils.AnnotationUtils | static |
| getParents(T child, Map< String, Collection< T >> parents) | umms.core.utils.AnnotationUtils | static |
| getSubAnnotationMidpointWithinAnnotation(Annotation annot, Annotation subAnnot) | umms.core.utils.AnnotationUtils | static |
| loadAnnotations(String bedFile) | umms.core.utils.AnnotationUtils | static |
| logger | umms.core.utils.AnnotationUtils | static |
| mapChildToLargestParent(Map< String, Collection< T >> children, Map< String, Collection< T >> parents) | umms.core.utils.AnnotationUtils | static |
| mapChildToParents(Map< String, Collection< T >> children, Map< String, Collection< T >> parents) | umms.core.utils.AnnotationUtils | static |
| mapParentsToChildren(Map< String, Collection< T >> children, Map< String, Collection< T >> parents) | umms.core.utils.AnnotationUtils | static |
| mergeOverlappingBlocks(TreeSet<?extends Annotation > windows) | umms.core.utils.AnnotationUtils | static |
| mergeOverlappingBlocksSingleOrientation(Collection< T > regions, boolean enforceSameOrientation) | umms.core.utils.AnnotationUtils | static |
| pairwiseNonoverlappingIgnoreStrand(Collection< Annotation > regions) | umms.core.utils.AnnotationUtils | static |


---

Generated on Fri Aug 15 2014 15:11:15 for ESAT by  

 1.8.7
